# Proof of concept for quantitative adverse outcome pathway modeling of chronic toxicity in repeated exposure

**DOI:** 10.1038/s41598-024-55220-4

**Published:** 2024-02-27

**Authors:** Shigeaki Ito, Sayak Mukherjee, Kazuo Erami, Shugo Muratani, Akina Mori, Sakuya Ichikawa, William White, Kei Yoshino, Dawn Fallacara

**Affiliations:** 1 Scientific Product Assessment Center, Japan Tobacco Inc., 6-2, Umegaoka, Aoba-ku, Yokohama, Kanagawa 227-8512 Japan; 2 Battelle, 505 King Ave., Columbus, OH 43201 USA

**Keywords:** Computational biology and bioinformatics, Computational models, Probabilistic data networks

## Abstract

Adverse Outcome Pathway (AOP) is a useful tool to glean mode of action (MOE) of a chemical. However, in order to use it for the purpose of risk assessment, an AOP needs to be quantified using in vitro or in vivo data. Majority of quantitative AOPs developed so far, were for single exposure to progressively higher doses. Limited attempts were made to include time in the modeling. Here as a proof-of concept, we developed a hypothetical AOP, and quantified it using a virtual dataset for six repeated exposures using a Bayesian Network Analysis (BN) framework. The virtual data was generated using realistic assumptions. Effects of each exposure were analyzed separately using a static BN model and analyzed in combination using a dynamic BN (DBN) model. Our work shows that the DBN model can be used to calculate the probability of adverse outcome when other upstream KEs were observed earlier. These probabilities can help in identification of early indicators of AO. In addition, we also developed a data driven AOP pruning technique using a lasso-based subset selection, and show that the causal structure of AOP is itself dynamic and changes over time. This proof-of-concept study revealed the possibility for expanding the applicability of the AOP framework to incorporate biological dynamism in toxicity appearance by repeated insults.

## Introduction

Regulatory agencies such as the U.S. Environmental Protection Agency (EPA), the U.S. Food and Drug Administration (FDA), European Chemical Agency (ECHA), and others are adopting the use of new approach methodologies (NAMs) to reduce animal use as part of their 3Rs (refinement, reduction, and replacement) agenda^1^. Fit-for-purpose in vitro NAMs are now used to interrogate a “system” at different layers of biological organization, from molecular to phenotypical. Integrating all these information together into a cohesive predictive model of an adverse outcome however is not so easy.

One solution to this problem is the adverse outcome pathway (AOP)^2,3^ framework. AOP framework proposes a simplification of holistic and systemic approaches where an apical adverse outcome (AO) is causally linked to an early molecular initiating event (MIE), like a receptor binding or production of reactive oxygen species, by subsuming hundreds and thousands of intermediate biological processes, from molecular, cellular, to organism level, into a handful of causally linked intermediate key events (KEs).

Although the AOP framework is expected to be a crucial tool for regulatory decision-making, given the qualitative nature of AOPs, direct application in quantitative risk assessment is challenging. This limitation highlights the need for quantitative AOP (qAOP) to bridge the gap between qualitative AOPs and the quantitative requirements of risk assessment. To circumvent this difficulty, several mathematical approaches were proposed and reviewed to render AOPs quantitative (qAOP) starting from simple dose–response modeling to more complicated Bayesian network modeling and differential equation (DE) based Systems Biology approaches^4–6^. Bayesian network (BN) formalism gained popularity as it can harmonize different types of data, provide a robust paradigm for causal modeling, and can be used prospectively for exploring multiple hypothesis. BNs have been used in studies spanning reproductive toxicity, developmental neural toxicity, cardiotoxicity, kidney injury etc.^4,5,7–12^ (the others can be found in Spinu’s article in 2020). In particular, Zgheib et al. reported several options for qAOP modeling approach for chronic toxicity, in which they applied Dynamic Bayesian Network (DBN) approach for chronic kidney disease by adaptation of time-series in vitro data into the qAOP model^13^. However, appearance of chronic toxicity/disease is often more complex, especially those associated with phenotypical changes in tissues or organ level from chronic exposure to low doses of chemical. For example, very low-level exposure to cadmium is not a serious concern as cadmium is contained in food to some extent. However, repeated (chronic) exposure to even low dose of cadmium can cause severe health impacts^14^. Cigarette smoking is a known risk factor for chronic diseases, but a single puff is hardly a health risk. As such, while an adverse outcome typically arise following the rapid biological responses triggered by certain reactive chemicals, there are the others that require cumulative biological reactions elicited by repeated and chronical exposure.

With advancement of in vitro NAMs, it is now feasible to perform experiments that can probe chronic toxic endpoints from repeated exposures^15–19^. Such technological innovations make it possible to expand the scope of the AOP framework: AOP which consist of acute phase responses and chronic phase response could be now realistic application for risk assessment strategy if appropriate risk quantification method is adopted.

**Figure 1:**
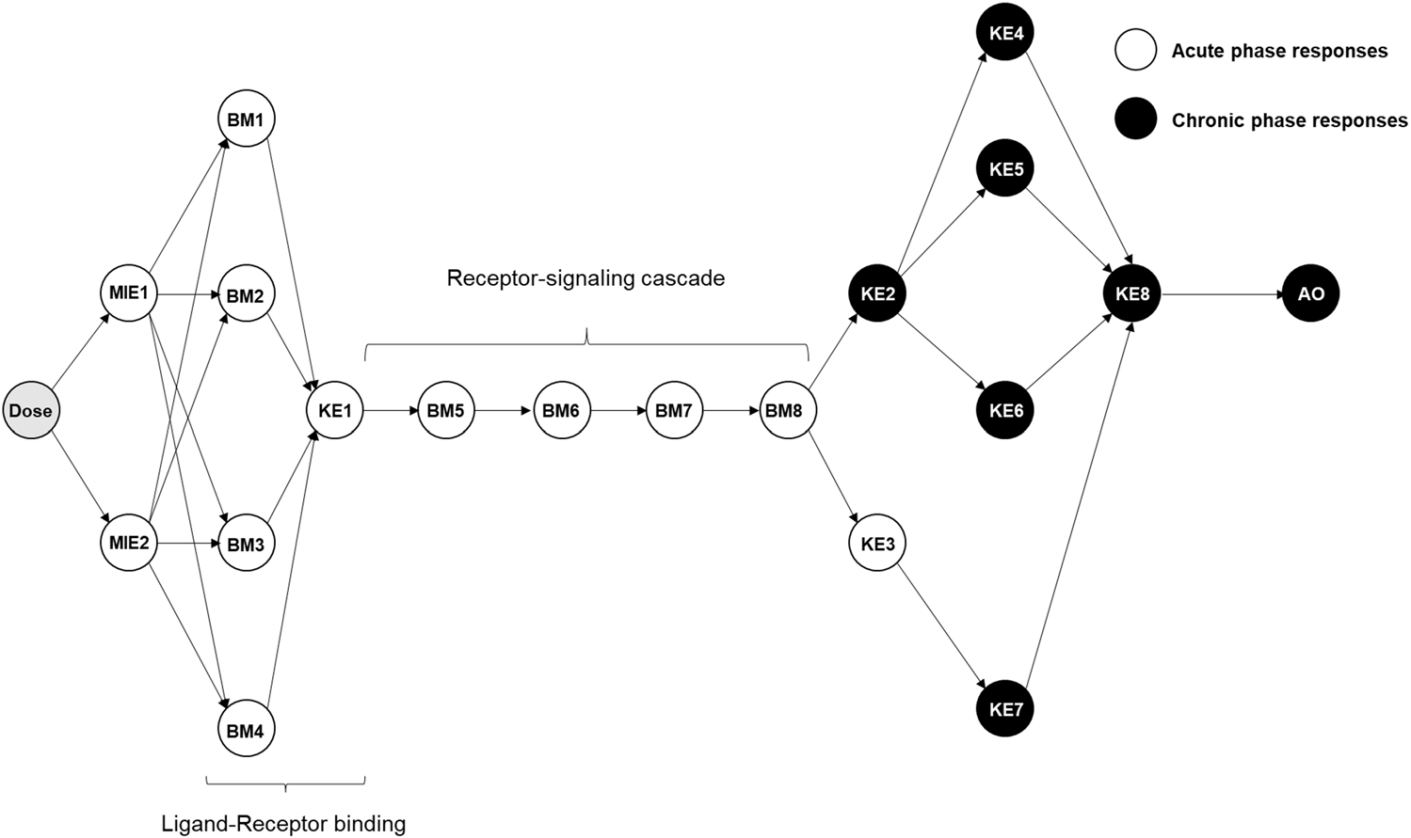
Directed acyclic graph of hypothetical AOP. Directed acyclic graph was used for Bayesian statistics-based approaches. BM1 through BM4 are ligands of the receptor (KE1). KE1 induces receptor signaling cascades of BM5 through BM8, which results in the downstream causal responses including chronic phase KEs. MIEs, acute-phase KEs, and acute-phase BMs are represented in white. Chronic phase KEs and AO are shown in solid black circles. MIE; Molecular initiating event, KE; Key event, BM; Biomarker, AO; adverse outcome.

Here, as a proof of concept, we developed a “hypothetical” AOP that has two distinct but interconnected modules, one for acute phase KEs and the other of chronic phase KEs (Fig. 1). We assumed that the chronic phase KEs can be elicited only after repeated/chronic exposures to an agent. Furthermore, in the context of repeated exposures, there is individual variation in the timing of manifestation of chronic toxicity, much like in vivo, where the onset of chronic toxicity may vary among donors of primary cells even under the same repeated exposure. To reflect this, in this proof-of-concept study, the timing of chronic toxicity appearance is varied for each donor. We used virtual data—generated using realistic assumptions—for this proof-of-concept study and quantified the AOP using a combination of static and dynamic Bayesian network formalisms (BN and DBN respectively). We show that the probabilities of adverse outcome can be estimated based on activation of upstream KEs earlier. We also use a data driven AOP pruning approach to quantify the dynamic nature of causal links in an AOP, as well as how these links evolve as a function of repeated insults.

## Methods

### Notation convention

Exposure repetition is indexed by $e=\mathrm{1,2},.,.,E$. Donors are denoted by $n=\mathrm{1,2},.,., N$. The dose (i.e., exposure concentration) is denoted by $d\in D$, where $D$ is the set of all doses including non-treatment control. MIEs, KEs, BMs, and AO are collectively referred to as nodes and denoted by $v\in V$, where $V$ is the set of all nodes. The total number of elements in a set is denoted by |..|; $\left|V\right|$ for example, stands for total number of nodes. Replicates for each donor will be denoted by $r=\mathrm{1,2},.,.,\mathcal{R}$. Matrices are written in double struck upper case letters: ${\mathbb{X}}_{M\times \left|V\right|}$ is a matrix with M rows and $\left|V\right|$ columns. Furthermore, a symbol such as ${\mathbb{X}}^{\left(e\right)}$ means a matrix ${\mathbb{X}}$ for a specific exposure repetition *e*. Similarly, ${\mathbb{X}}^{\left(e,n\right)}$ is understood as a matrix for a specific exposure repetition *e* and a donor $n$. Elements of a matrix and scalars in general are written in lower-case fonts. A vector is always a column vector and denoted in bold: ${\varvec{\beta}}\in {R}^{P}$ is a vector with *P* components. A one vector, ${1}_{P\times 1}\in {R}^{P}$, is a column vector of $P$ ones. Transpose of a matrix or a vector is denoted by the capital letter $T$ in the superscript. A node $v$ is assumed to take continuous values and in Bayesian formalism, the probability that it takes a value *x* depends only on the values of its parents, i.e. $P\left({x}^{(\nu )} | {\varvec{x}}\in {R}^{V}\right)=P\left({x}^{(\nu )} | {{\varvec{x}}}^{({\Pi }_{v})}\in {R}^{{|\Pi }_{v}|}\right)$, where ${\Pi }_{v}$ denotes the set of parents of node $v$.

### Software and algorithms

We used Microsoft Excel for generation of the primary dataset. All the other analyses and visualization were performed using R statistics software version 4.0.4. The algorithms and used R packages are summarized in the Supplementary material 1.

### Structure of hypothetical AOP

The network structure is shown in Fig. 1. To model chronic toxicity, we constructed a hypothetical AOP with two MIEs, two acute-phase KEs, eight acute-phase biomarkers (BMs), six chronic-phase KEs, and an AO. Activation of some biomarkers, such as ligands for receptor-ligands binding, and activation of downstream signaling molecules for signal transduction are crucial for elicitation of AO. We included them in our AOP and denoted them as BMs. While BMs are usually not included in AOPs, we included them in this proof-of-concept study to highlight the fact that additional assays that capture relevant endpoints upstream of or related to a specific KE are usually obtained in a lab. For example, one may collect activation of biomarkers in MAP kinase pathway upstream of any apoptotic KE. Moreover, from a modeling point of view, any number of BMs can be included in the modeling if their wiring does not violate the fundamental assumption of Bayesian Network analysis, namely generation of feedback loops and cycles. To keep the discussion as general as possible, biomarkers that lead to KEs are also included in the model.

Specifically, BM1 through BM4 mimic ligands for KE1 (e.g., receptor activation), and BM5 through BM8 represents the downstream signal transduction cascade. KE2 and KE3 represent the products of signal transduction, but KE2 only appears in chronic phase, while KE3 appears upon every exposure. Once chronic-phase response appears, a series of chronic-phase responses simultaneously appear as a chain reaction. We have collectively referred to them as nodes (19 nodes in total). In this AOP, we assumed that twelve biological events including MIEs, acute-phase KEs and biomarkers were elicited in a dose dependent manner for every exposure repetition. Chronic-phase KEs were also elicited in dose dependent manner but only after being subjected to a given number of exposure repetition. Specific timing of a chronic phase KE varied across donors (Table 1).

**Table 1:** Assumption of donor dependent appearance of chronic toxicity.

Donor	Appearance of chronic toxicity	Severity
1	Exposure rep 4	Severe
2	Exposure rep 3	Severe
3	Exposure rep 4	Moderate
4	Exposure rep 3	Moderate
5	Exposure rep 4	Weak
6	Exposure rep 3	Weak
7	No	No
8	No	No

### Primary virtual date generation

For this proof-of-concept study, we used a fully virtual dataset rather than actual data, because there are hardly any available data appropriate for chronic toxicity related repeated exposure based qAOP modeling.

The assumptions and parameters used for the primary virtual dataset are:

i. Four doses including non-treated control ($\left|D\right|=4$), total number of donors $N=8$, and the number of exposures $E=6$
ii. Acute-phase biological responses (i.e., MIEs, KE1, KE3, and BM1 through BM8) show robust dose-dependence for all exposures.
iii. The timing of appearance of chronic-phase responses (i.e., KE2 and KE4 through KE8, and AO) are donor-dependent (Table 1).
iv. Once elicited, chronic phasae responses also increase in a dose-dependent manner. They also exhibit a robust exposure-repetition dependence upon elicitation.
v. Denoting the replicate average and standard deviation of fold change at a dose *d* by ${\overline{{\varvec{f}}} }^{(d)}$ and ${{\varvec{s}}}^{(d)}$, we assumed that ${{\varvec{s}}}^{(d+1)}$ is affected by the difference in ${\overline{{\varvec{f}}} }^{(d)}$ and ${\overline{{\varvec{f}}} }^{(d+1)}$: larger the difference, larger is ${{\varvec{s}}}^{(d+1)}$.
vi. The fold-changes follow a log-normal distribution.

First, the *mean fold-changes* for all nodes for $d=0$ (air control) were set to 1, i.e., ${\overline{f} }^{\left(\nu ,d=0,n\right)}=1 \forall \nu ,n$. Mean fold-changes for subsequent doses and exposures for all *acute phase nodes* (MIE, KE1, KE3, and all BMs) were then generated using the formulae ${\overline{f} }^{(d+1,\nu ,n,e)}={\overline{f} }^{(d,\nu ,n,e)}+{\zeta }_{d}$ and ${\overline{f} }^{(e+1,\nu ,n,d)}={\overline{f} }^{(e,\nu ,n,d)}+{\zeta }_{e}$ respectively, where ${\overline{f} }^{(d,e,n,\nu )}$ stands for mean fold-change of node $\nu$ of donor *n* at dose *d,* exposure *e*, and ${\zeta }_{d}\in U(\mathrm{0,1})$ and ${\zeta }_{e}\in U\left(\mathrm{0,0.2}\right)$ are uniform random numbers. Denoting onset time of a chronic response for a donor *n* by ${e}_{ch}^{n}$ (Table 1), mean fold change for chronic phase KEs were generated using the formulae above for exposure repetitions $e>{e}_{ch}^{n}$. For $e<{e}_{ch}^{n}$, the mean fold changes for the chronic phase KEs were randomly populated using the formula $0.8+0.4\zeta \in U(\mathrm{0,1})$, thereby making sure that they were not elicited. Similarly, standard deviation of acute phase biomarkers for all exposures and chronic phase biomarkers for $e>{e}_{ch}^{n}$ were generated using the formula ${s}^{(d+1,\nu ,n,e)}=\left({\overline{f} }^{(d,\nu ,n,e)}+\zeta \times \left({\overline{f} }^{(d+1,\nu ,n,e)}-{\overline{f} }^{(d,\nu ,n,e)}\right)\right)/\left(4+6\zeta \right)$, where ${s}^{(d+1)}$ is the standard deviation of fold-change for that dose.

The mean fold changes of each node as functions of dose and exposure repetition for all $N=8$ donors are summarized in Supplementary material 2.

### Consideration of correlation co-efficient for resampling the primary dataset

For each donor, dose, and exposure repetition, we log transformed the mean fold changes and constructed the *mean log-fold change* vector ${{\varvec{\mu}}}^{\left(n,e,d\right)}\in {R}^{\left|V\right|}$ such that its components ${\mu }^{\left(n,e,d,\nu \right)}, v=\mathrm{1,2},.,\left|V\right|$, were given by the formula

1 $$\begin{array}{c}{\mu }^{\left(n,e,d,v\right)}=ln\left(\frac{{\left({\overline{f} }^{\left(n,e,d,v\right)}\right)}^{2}}{\sqrt{{\left({\overline{f} }^{\left(n,e,d,v\right)}\right)}^{2}+{\left({s}^{\left(n,e,d,v\right)}\right)}^{2}}}\right)\\ \end{array}.$$

We know that biological responses elicited by a common stimulus are usually correlated with each other, especially when they show clear dose responses. Therefore, we accounted for response-response correlation at the donor level for each exposure repetition. Correlations were calculated from the primary dataset. The elements of response-response covariance matrix ${\mathbb{O}}_{\left|V\right|\times \left|V\right|}^{\left(n,e,d\right)}$, were given by

2 $$\begin{array}{c}{o}_{v{v}^{\prime}}^{\left(n,e,d\right)}={\left({\sigma }^{\left(n,e,d,v\right)}\right)}^{2}=ln\left(1+\frac{{\left({s}^{\left(n,e,d,v\right)}\right)}^{2}}{{\left({\overline{f} }^{\left(n,e,d,v\right)}\right)}^{2}}\right) for v={v}^{\prime}\\ {o}_{v{v}^{\prime}}^{\left(n,e,d\right)}={\rho }_{v{v}^{\prime}}^{\left(e\right)}\sqrt{{o}_{vv}^{\left(n,e,d\right)}*{o}_{{v}^{\prime}{v}^{\prime}}^{\left(n,e,d\right)}} for v\ne {v}^{\prime}\end{array},$$

where the correlation co-efficient ${\rho }_{v{v}^{\mathrm{^{\prime}}}}^{\left(e\right)}={\sigma }_{v{v}^{\mathrm{^{\prime}}}}^{\left(e\right)}/\sqrt{{\sigma }_{vv}^{\left(e\right)}\times {\sigma }_{{v}^{\mathrm{^{\prime}}}{v}^{\mathrm{^{\prime}}}}^{\left(e\right)}}$.

Using Eq. (1) and Eq. (2), we drew samples from a multivariate normal distribution $\mathcal{M}\mathcal{N}\left({{\varvec{\mu}}}^{\left(n,e,d\right)},{\mathbb{O}}_{\left|V\right|\times \left|V\right|}^{\left(n,e,d\right)}\right)$ for each donor, exposure repetition, and dose.

### Bayesian updating approach for resampling the primary dataset

In addition, we considered the possibility that the data at a previous exposure repetition could be informative for the next exposure repetition. To account for that, we explicitly introduced causation between each node with a Gaussian Bayesian network (GBN) update scheme.

Commonly, parameter learning of a GBNs entails maximizing a Gaussian likelihood of the form $\mathcal{M}\mathcal{N}\left({{\varvec{x}}}^{\left(\nu \right)}|{\mathbb{X}}_{M\times \left|{\Pi }_{v}\right|}{\varvec{\beta}}+{\beta }_{0},{\sigma }^{2}{\mathbb{I}}\right)$, where ${\Pi }_{v}$ is the parent set of a node $\nu$, $M$ is the number of samples, ${\sigma }^{2}$ is the residual, and $\left({\varvec{\beta}},{\beta }_{0}\right)$ are the parameters to be estimated. Maximum Likelihood Estimate (MLE) for a Gaussian likelihood is simply ${\widehat{{\varvec{\beta}}}}_{{\varvec{O}}{\varvec{L}}{\varvec{S}}}={\left({{\mathbb{X}}_{M\times {\Pi }_{v}}}^{T}{\mathbb{X}}_{{M\times \Pi }_{v}}\right)}^{-1}{{\mathbb{X}}_{M\times {\Pi }_{v}}}^{T}{{\varvec{x}}}^{\left(\nu \right)}$ (ordinary linear regression estimates). Alternatively, we can estimate ${\varvec{\beta}}$ by using a Gaussian prior of the form $\mathcal{M}\mathcal{N}\left({\varvec{\beta}}|{{\varvec{\beta}}}_{{\varvec{p}}{\varvec{r}}},{\mathbb{V}}_{pr}\right)$. When ${\sigma }^{2}$ is known, the posterior of ${\varvec{\beta}}$ is a multivariate Gaussian $\mathcal{M}\mathcal{N}\left({\varvec{\beta}}|{\widehat{{\varvec{\beta}}}}_{pos},{\mathbb{V}}_{pos}\right)$, where

3 $$\begin{array}{c}{\widehat{{\varvec{\beta}}}}_{pos}={\mathbb{V}}_{pos}\left({{\mathbb{V}}_{pr}}^{-1}{{\varvec{\beta}}}_{{\varvec{p}}{\varvec{r}}}+\frac{1}{{\sigma }^{2}}{{\mathbb{X}}_{{M\times \Pi }_{v}}}^{T}{{\varvec{x}}}_{v}\right)\\ {\mathbb{V}}_{pos}^{-1}={{\mathbb{V}}_{pr}}^{-1}+\frac{1}{{\sigma }^{2}}{{\mathbb{X}}_{M\times {\Pi }_{v}}}^{T}{\mathbb{X}}_{{M\times \Pi }_{v}}\end{array}.$$

First, for each exposure $e$, dose $d$ and donor $n$, we drew $\kappa$ samples ${\mathbb{X}}_{\kappa \times \left|V\right|}^{(n,e,d)}\sim \mathcal{M}\mathcal{N}\left({{\varvec{\mu}}}^{\left(n,e,d\right)},{\mathbb{O}}_{\left|V\right|\times \left|V\right|}^{\left(n,e,d\right)}\right)$. Then, we learned the parameters ${{\widehat{{\varvec{\beta}}}}^{\left(n,e,d\right)}}_{pos}$ and ${{\mathbb{V}}^{\left(n,e,d\right)}}_{pos}$ iteratively using ${\mathbb{X}}_{{\kappa \times \Pi }_{v}}$ (relevant sub-matrix of ${\mathbb{X}}_{\kappa \times \left|V\right|}^{(n,e,d)}$) and Eq. (3) by replacing ${{\varvec{\beta}}}_{{\varvec{p}}{\varvec{r}}}$ and ${\mathbb{V}}_{pr}$ in Eq. (3) with ${{\widehat{{\varvec{\beta}}}}^{\left(n,e-1,d\right)}}_{pos}$ and ${{\mathbb{V}}^{\left(n,e-1,d\right)}}_{pos}$ respectively. For the *e* = *0*, OLS estimate for ${\varvec{\beta}}$ was used. Usually, ${\sigma }^{2}$ is unknown. Prior over ${\sigma }^{2}$ (Inverse Wishart distribution) can be used and standard result can be applied. Instead, we pretended to know ${\sigma }^{2}$ by setting it to the ordinary least square estimate

4 $${\left({\widehat{\sigma }}^{\left(n,e,d\right)}\right)}^{2}=\frac{{{{\varvec{x}}}^{\left(\nu \right)}}^{T}\left({{\varvec{x}}}^{\left(\nu \right)}-{\mathbb{X}}_{\kappa \times \left|{\Pi }_{v}\right|}^{\left(n,e,d\right)}{{\widehat{{\varvec{\beta}}}}^{({\varvec{e}})}}_{{\varvec{O}}{\varvec{L}}{\varvec{S}}}\right)}{\kappa -\left|{\Pi }_{v}\right|}.$$

Please note that we explicitly used the causal structure of the AOP (Fig. 1) for estimating these parameters.

Subsequently, we constructed a virtual dataset ${\mathbb{X}}_{\mathcal{R}\times \left|V\right|}^{(n,e,d)}$ by resampling from this trained network using the standard formula $P\left({{{\varvec{x}}}^{\left(\nu \right)}}_{pred}|{\mathbb{X}}^{\left(test\right)},{\sigma }^{2}{\mathbb{I}}\right)=\mathcal{M}\mathcal{N}\left({{{\varvec{x}}}^{\left(\nu \right)}}_{pred}|{\mathbb{X}}^{\left(test\right)}{\widehat{{\varvec{\beta}}}}_{pos},{\sigma }^{2}{\mathbb{I}}+{\mathbb{X}}^{\left(test\right)}{\mathbb{V}}_{pos}{\left({\mathbb{X}}^{\left(test\right)}\right)}^{T}\right)$. Replicate averaged virtual data ${{\varvec{x}}}^{(n,e,d)}=\left({\left({X}_{\mathcal{R}\times \left|V\right|}^{(n,e,d)}\right)}^{T}1\right)/\mathcal{R}\in {R}^{V}$ was used for all downstream analysis.

### Dose–response and response-response analysis

We performed dose–response analysis using a linear mixed model (LMM) with a donor specific random effect of the form

5 $${{\varvec{x}}}^{\left(\nu ,n,e\right)}={\beta }_{0}{1}_{\left|D\right|\times 1}+{\beta }_{1}{{\varvec{d}}}_{\left|D\right|\times 1}+{\gamma }^{\left(n\right)}{1}_{\left|D\right|\times 1}+\varepsilon ,$$

where ${\gamma }^{\left(n\right)}\sim \mathcal{N}\left(0,{\sigma }_{n}^{2}\right)$ is the random effect on the intercept. ${\varvec{d}}$ is the vector of doses, and ${{\varvec{x}}}^{\left(\nu ,n,e\right)}$ is a replicate averaged fold-change vector for a given node $\nu$, donor $n$, and exposure repetition $e$.

For response-response relationships, we used two models, a) LMM model with donor specific random effect on the intercept, and b) a cubic polynomial model of forms

6 $$\left.\begin{array}{c}{{\varvec{x}}}^{\left(\nu ,n\right)}=\left({\beta }_{0}+{\gamma }^{\left(n\right)}\right){1}_{\left|D\right|*E\times 1}+{\beta }_{1}{{\varvec{x}}}^{\left({\nu }^{\prime},n\right)}+\varepsilon \\ {{\varvec{x}}}^{\left(\nu \right)}={\beta }_{0}{1}_{\left|D\right|*E*N\times 1}+{\beta }_{1}{{\varvec{x}}}^{\left({\nu }^{\prime}\right)}+{\beta }_{2}{\left[{{\varvec{x}}}^{\left({\nu }^{\prime}\right)}\right]}^{2}+{\beta }_{2}{\left[{{\varvec{x}}}^{\left({\nu }^{\prime}\right)}\right]}^{3}+\varepsilon \end{array}\right\},$$

where ${\left[{{\varvec{x}}}^{\left({\nu }^{\prime}\right)}\right]}^{2}$, ${\left[{{\varvec{x}}}^{\left({\nu }^{\prime}\right)}\right]}^{3}$ imply element wise exponentiation. Owing to large number of possible pairs $\left(\nu ,{\nu }^{\prime}\right)$, we did not analyze the data separately for each exposure repetition, instead analyzed the combined dataset for all exposure repetition.

### Bayesian modeling and probability calculation of AO

Here, we looked at each exposure repetition separately. The underlying assumption here is that the time gap between successive exposures is much longer compared to the time scale associated with the emergence of the AO. Clearly, for chronic conditions, this assumption is not true. Yet, we modelled this hypothetical scenario as a proof-of-concept for acute conditions where such an assumption may find more merit. We used a GBN—a directed acyclic graph (DAG)$\mathcal{G}=\left(V,E\right)$, where $V$ is the set of nodes and $\mathcal{E}$ is the set of edges between nodes in $V$. A DAG can be topologically ordered such that the edges point from topological ancestors to their successors. In Bayesian formalism, the joint probability of a realization ${\varvec{x}}\in {R}^{\left|V\right|}$ can be factorized as follows

7 $$P\left({\varvec{x}}\right)=\prod_{\nu =1}^{\left|V\right|}P\left({x}^{\left(\nu \right)} | {{\varvec{x}}}^{\left({\Pi }_{v}\right)}\right),$$

where ${\Pi }_{\nu }$ is the set of parents of node $\nu$, and ${{\varvec{x}}}^{\left({\Pi }_{v}\right)}$ is the value that they take. The likelihood $P\left({x}^{\left(\nu \right)} | {{\varvec{x}}}^{\left({\Pi }_{v}\right)}\right)$ is given by $P\left({x}^{\left(\nu \right)} | {{\varvec{x}}}^{\left({\Pi }_{v}\right)}\right)=\mathcal{N}\left({x}^{\left(\nu \right)}|{{{\varvec{x}}}^{\left({\Pi }_{v}\right)}}^{T}{\varvec{\beta}},{\sigma }^{2}\right)$ where unknown parameter ${\varvec{\beta}}$ is learned by fitting a linear regression model^20,21^.

The DAG of the hypothetical AOP is shown in Fig. 1. Dose was included as root node without parent nodes. Dose was assumed to be a continuous variable. The parameters of the model were learned by fitting the AOP to ${\mathbb{X}}^{\left(e\right)}$ (data for each exposure repetition) using a function from the bnlearn package^22^ called bn.fit. The fitted parameter values are summarized in Supplementary tables 1. The trained GBNs were resampled to estimate the probability of activation of KEs ($P\left(KE>\Delta |d\in \left[d\pm \varepsilon \right]\right)$) for each exposure repetition using logic sampling. A typical ${\Delta =\mathit{log}}_{10}2$ was chosen. We also varied $\Delta$ and the dose *d* to investigate how this probability changed as a function of *d* and $\Delta$. We also calculated the volume under the probability surface (VUS) using standard trapezoid rule.

### Dynamic Bayesian modeling and probability of adverse outcome conditioned on activation of upstream chronic events at earlier exposure repetitions.

A dynamic Bayesian model (DBN) was developed to estimate transition probabilities of AO by conditioning it on the upstream events at a previous exposure repetition ($e-\tau$) using a multivariate Markov process of the form^23^.

8 $${{\varvec{x}}}^{\left(e,\nu \right)}={\mathbb{X}}^{\left(e-1,{\Pi }_{\nu }\right)}{{\varvec{\beta}}}^{\left(e,\nu \right)}+{{\varvec{\varepsilon}}}^{\left({\varvec{e}},\nu \right)}\sim \mathcal{N}\left(0,{\sigma }^{2}{\mathbb{I}}\right)$$

**Figure 2:**
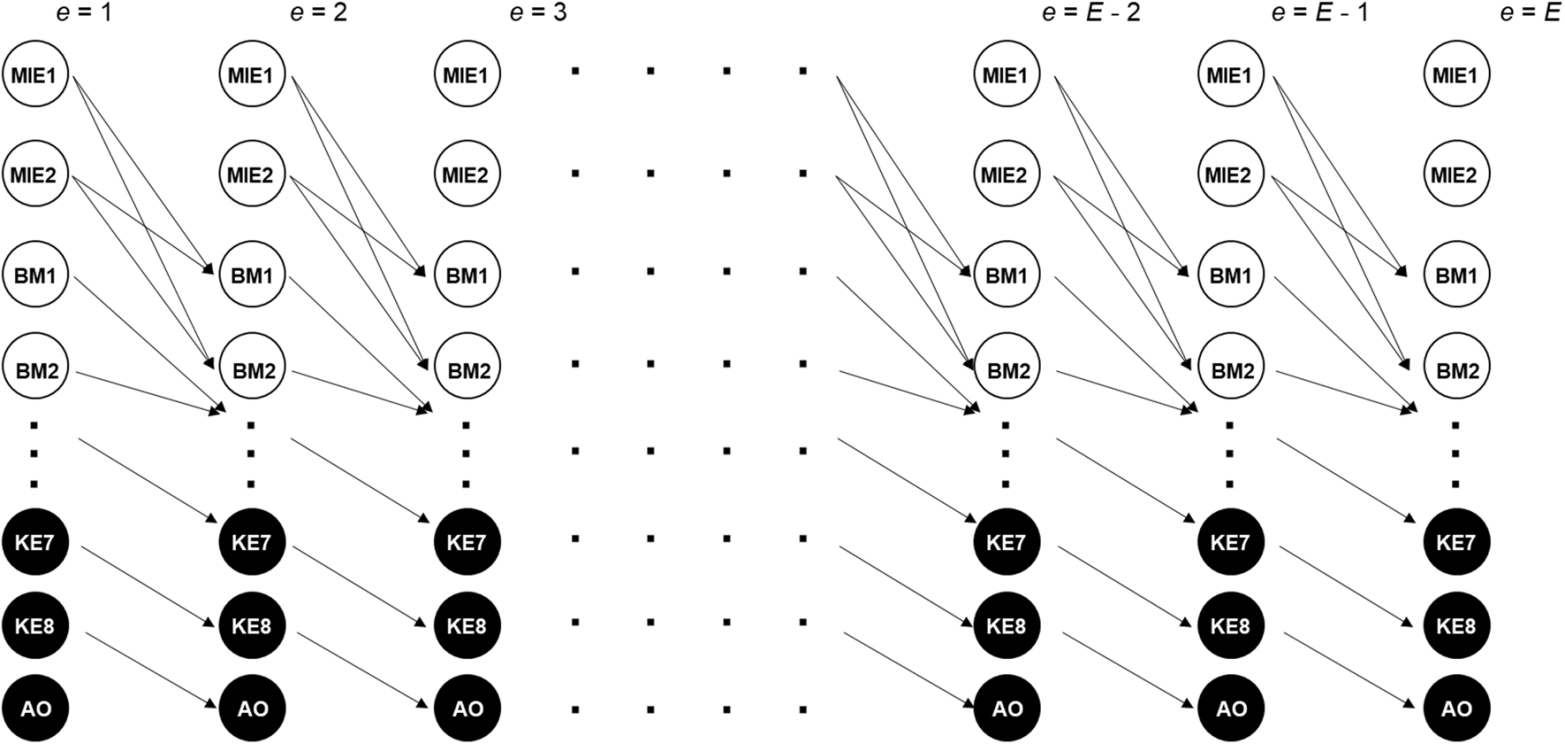
Diagrammatic representation of Dynamic Bayesian Network of the AOP. The nodes are causally connected across exposure repetitions, where a parent node at a previous exposure can influence the child node at a later exposure. We did not include any causal link between nodes at a given exposure. We assumed a first-order Markov process ${\varvec{x}}^{\left( e \right)} \leftarrow {\varvec{x}}^{{\left( {e - 1} \right)}}$ and did not assume stationarity.

Diagrammatic representation of our logic and structure of DBNs are shown in Fig. 2. In brief, we assumed that events at a previous exposure causally influenced events at the current exposure. For example, amplitude of KE6 at *e* = 4 was causally influenced by its parent node, i.e., amplitude of KE2 at *e* = 3, who was in turn influenced by its parent node, BM8, at *e* = 2, so and so forth. We further assumed that no causal links exist among nodes for the same exposure repetition. Formally, nodes at a given exposure *e* were conditionally independent of each other when their values at the previous exposure were known. Furthermore, we have assumed that values at present were only conditionally determined by values in the immediate past, i.e., ${\varvec{x}}^{\left( e \right)} \leftarrow {\varvec{x}}^{{\left( {e - 1} \right)}}$ (Markov process), but not by remote pasts $e-2, e-3,$ etc. The last two assumptions were not essential, but they were necessary to avoid number of parameters from exploding and thereby rendering parameter learning unfeasible. In addition, dose was not included as a root node in this framework. We assumed that dose-MIE interaction, which for a vast majority of AOPs are ligand-receptor types of interaction, occurs at a timescale much faster compared to the time-gap between successive exposures. As a result, the dose was treated as a discrete variable, and subsequently, we either pooled all the doses together or examined each of the three doses separately.

Although time-series analysis typically assumes that the process in weakly stationary, we relaxed weak stationarity criteria as evidenced by the fact that our ${\varvec{\beta}}$ explicitly depended on the exposure repetition index $e$ (see Eq. 8) and needed to be learned for each pair of exposure slices (for example $, {{\varvec{\beta}}}^{\left(e=3\right)}\ne {{\varvec{\beta}}}^{\left(e=5\right)}$: ${{\varvec{\beta}}}^{\left(e=3\right)}$ was estimated using data from exposure slice $\left(e=2,e=3\right)$, whereas ${{\varvec{\beta}}}^{\left(e=5\right)}$ was estimated using data from exposure slice ($e=4,e=5$)). Moreover, we also observed very strong correlations between chronic phase responses (see Result section), which made straightforward interpretation of ordinary multivariate linear regression difficult. As subset selection algorithms would prune the causal links between nodes of AOP, we did not use them here. Instead, we used ridge regularization^24^. The parameters were learned using glmnet package^25^ and appropriate penalty ${\lambda }^{*}$ was selected using leave-one-out-cross-validation (LOOCV). For this exercise, we used an activation cut-off ${\Delta =\mathit{log}}_{10}2$. Combining data for all doses and donors, we numerically calculated **a)** $P\left({AO}^{\left(e\right)} > \Delta | {KE8}^{\left(e-1\right)} > \Delta \right)$ for $e=3,., 6$ (4 probabilities in total), **b)** $P\left({AO}^{\left(e\right)} > \Delta | {KE6}^{\left(e-2\right)} > \Delta \right)$, $P\left({AO}^{\left(e\right)} > \Delta | {KE5}^{\left(e-2\right)} > \Delta \right)$, $P\left({AO}^{\left(e\right)} > \Delta | {KE4}^{\left(e-2\right)} > \Delta \right)$, $P\left({AO}^{\left(e\right)} > \Delta | {KE7}^{\left(e-2\right)} > \Delta \right)$ for $e=4, 5, 6$ (3 probabilities in total for each KEs), and finally **c)** $P\left({AO}^{\left(e\right)} > \Delta | {KE2}^{\left(e-3\right)} > \Delta \right)$, $P\left({AO}^{\left(e\right)} > \Delta | {KE3}^{\left(e-3\right)} > \Delta \right)$ for $e=5, 6$ (2 probabilities in total for each KEs) by resampling data from DBNs and using likelihood weighing. We also calculated probabilities of AO for specific dose, $P\left({AO}^{\left(e,d\right)}>\Delta | {KE}^{\left(e-\tau ,d\right)}> \Delta \right)$, where the interaction strengths ${\varvec{\beta}}$ s were not only learned between successive exposure repetition slices but also for specific doses as shown below

9 $${{\varvec{x}}}^{\left(e,\nu ,d\right)}={\mathbb{X}}^{\left(e-1,{\Pi }_{\nu },d\right)}{{\varvec{\beta}}}^{\left(e,\nu ,d\right)}+{{\varvec{\varepsilon}}}^{\left({\varvec{e}},\nu ,d\right)}\sim \mathcal{N}\left(0,{\sigma }^{2}{\mathbb{I}}\right).$$

### Data driven pruning of AOP

As an additional exercise, we further implemented a data driven approach to restructure AOP, where we pruned AOP in Fig. 1 using a subset selection strategy: for nodes regulated by more than one predictor nodes, we implemented a LOOCV based lasso regularization that shrinks coefficients for uninformative/irrelevant predictors towards zero; for nodes that were regulated by one predictor, we only included it if the *p* value of the slope parameter was < 0.05. The customized BN was further scrutinized to only include those nodes for which at least one path existed to AO (for example, if the path from KE6 ⟶ KE8 had non-zero coefficient but KE8 ⟶ AO was zero, and since AO could not be reached from KE6 without visiting KE8, KE6 was pruned as well).

## Results

### Virtual data generation

The primary dataset was generated using random number generation function to suit the assumptions described earlier (see Methods section "Structure of hypothetical AOP"). Because there are only a few real-life datasets for qAOP modeling of chronic toxicity, we considered the primary dataset as the grand truth. The generated primary dataset is summarized in Supplementary material 2. For large number of replicates $r=\mathrm{1,2},.,.,\mathcal{R}$, the mean and the standard deviation of fold-change, $\sum_{r}{f}^{\left(n,e,d,v,r\right)}/\mathcal{R}$, $\sum_{r}{\left({f}^{\left(n,e,d,v,r\right)}-{\overline{f} }^{\left(n,e,d,v\right)}\right)}^{2}/\mathcal{R}$, would approach the mean fold-change (${\overline{f} }^{\left(n,e,d,v\right)}$) and standard deviation (${s}^{\left(n,e,d,v\right)}$) in the primary dataset. However, for small number of replicates (typically ~ 3), and small sample size effect can be significant. One way to account for small sample size is by means of confidence intervals. Alternately, one can re-sample a small number of samples $R\ll \mathcal{R}$ as discussed in the Methods section, and then take the replicate average of fold-change ${f}^{\left(n,e,d,v,r\right)}$. In this study, we resampled the primary dataset using Bayesian updating approach as described in the methods section "Consideration of correlation co-efficient for resampling the primary dataset"-"Bayesian updating approach for resampling the primary dataset". As expected, the virtually generated dataset showed that the chronic phase biomarkers were not readily activated, but after three successive exposure repetitions, moderate to high expression of chronic phase responses was observed (Supplementary Fig. 1).

### Dose–response and response-response relationship

**Figure 3:**
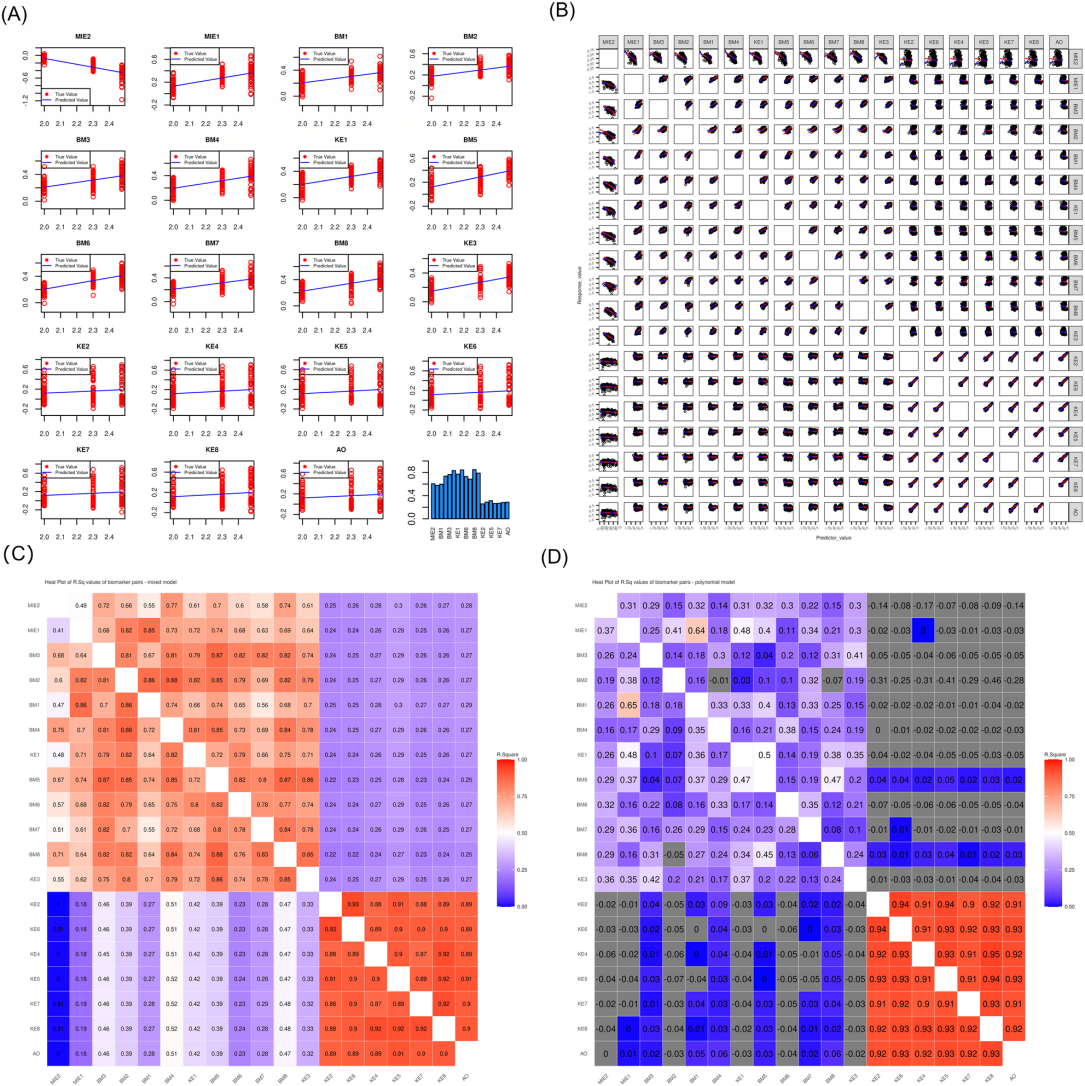
R^2^ values for dose–response and response-response models: Linear mixed model (LMM) was used for both dose–response (Eq. 5) and response-response modeling (Eq. 6a). The conditional R^2^ values^26^ were reported for both dose–response (**A**) and response-response modeling (**B**). For dose–response, each exposure repetition was analyzed separately. For response-response modeling, all exposure repetitions were pooled together. (**C**) Heatmap of R^2^ values of biomarker pairs. (**D**) Same as (**B**) and (**C**), except polynomial regression (Eq. 6b) was used and R^2^ coefficient was calculated using standard formula. These analyses and visualizations were performed using R statistics software version 4.0.4.

Figure 3 shows the conditional R^2^ values for dose–response and response-response modeling when LMM was used and standard training R^2^ value for the polynomial regression model. As expected, the acute phase nodes generally showed strong dose–response for all exposure repetitions, whereas the chronic phase nodes started to show dose response only for *e* $\ge$ 3. Response-response analysis was performed on the combined dataset for all exposure using a LMM and a polynomial regression model. We found that LMM outperformed polynomial regression model overall (Fig. 3), except for chronic-phase nodes, when they were comparable. Using analysis from LMM, we observed that all acute-phase nodes were highly correlated with each other. Similarly, all chronic-phase nodes were also correlated with each other; however, there was a low correlation between acute-phase nodes and chronic-phase nodes (Fig. 1C).

### Probability analysis with Bayesian modeling approach

**Figure 4:**
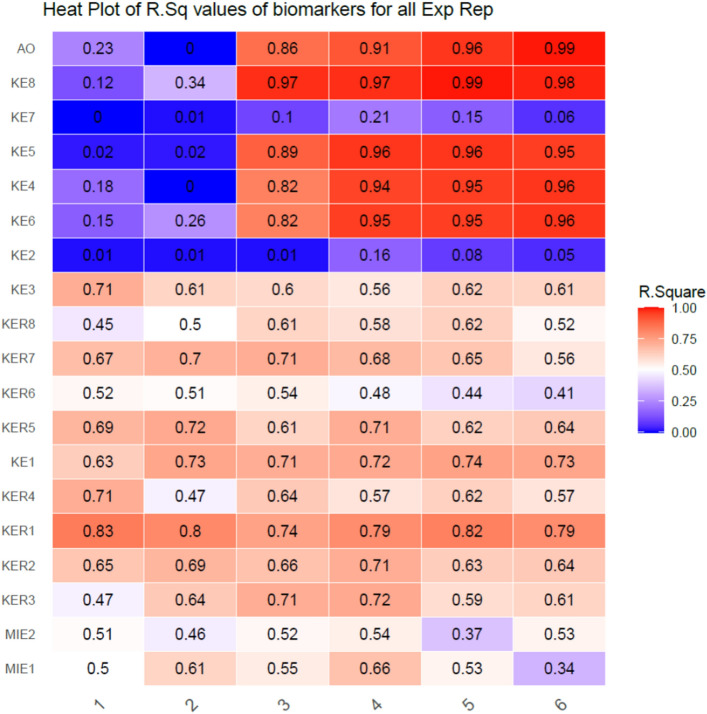
R^2^ of biomarkers for all exposure repetitions. R^2^ for each node was calculated using bnlearn that implements standard linear regression model. The predictors for each node were their parent set ${\Pi }_{\nu }$. The analysis and visualization were performed using R statistics software version 4.0.4.

GBNs were fitted to the data for each exposure repetition separately. Figure 4 shows the R^2^ values of those fits. Except for KE2 and KE7, R^2^ values of other chronic KEs were consistently > 0.6 for exposure repetition $e=3$ onwards. The fits for KE7 and KE2 did not do well for any exposure repetition because they were both regulated by upstream acute phase nodes KE3 and BM8 with whom they were very poorly correlated (Fig. 4). R^2^ values for other acute phase KEs were also > 0.6 by-n-large.

**Figure 5:**
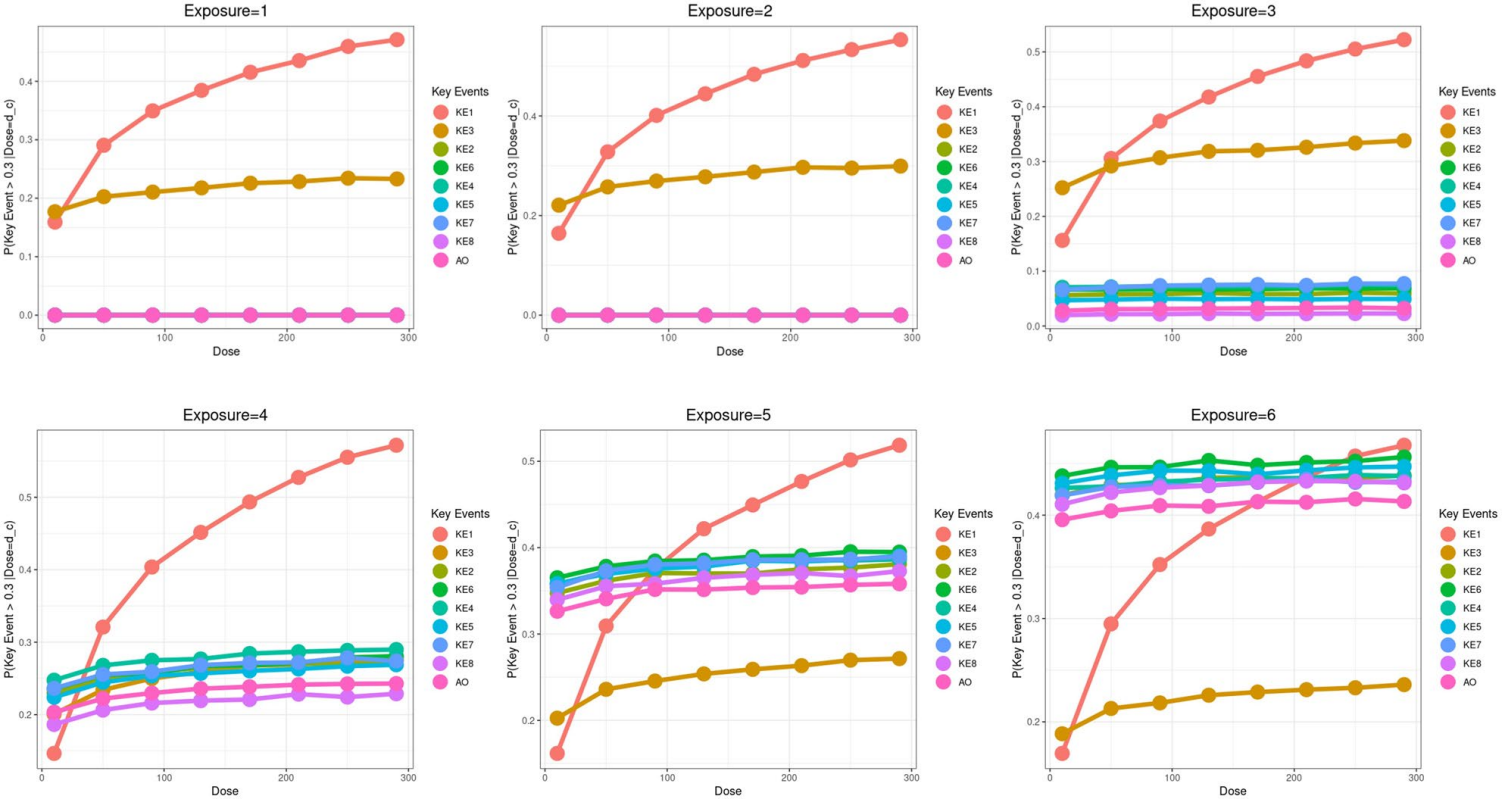
Dose-probability curves of Bayesian Network analysis: The probabilities were calculated by fitting the GBN to the virtual data, followed by logic sampling. The threshold of activation $\Delta$ was set to ${\mathit{log}}_{10}2$. The analysis and visualization were performed using R statistics software version 4.0.4. The lines corresponding to KE2, 4, 5, 6, 7, and 8 at the exposure 1 and 2 are hidden by the line of AO.

**Figure 6:**
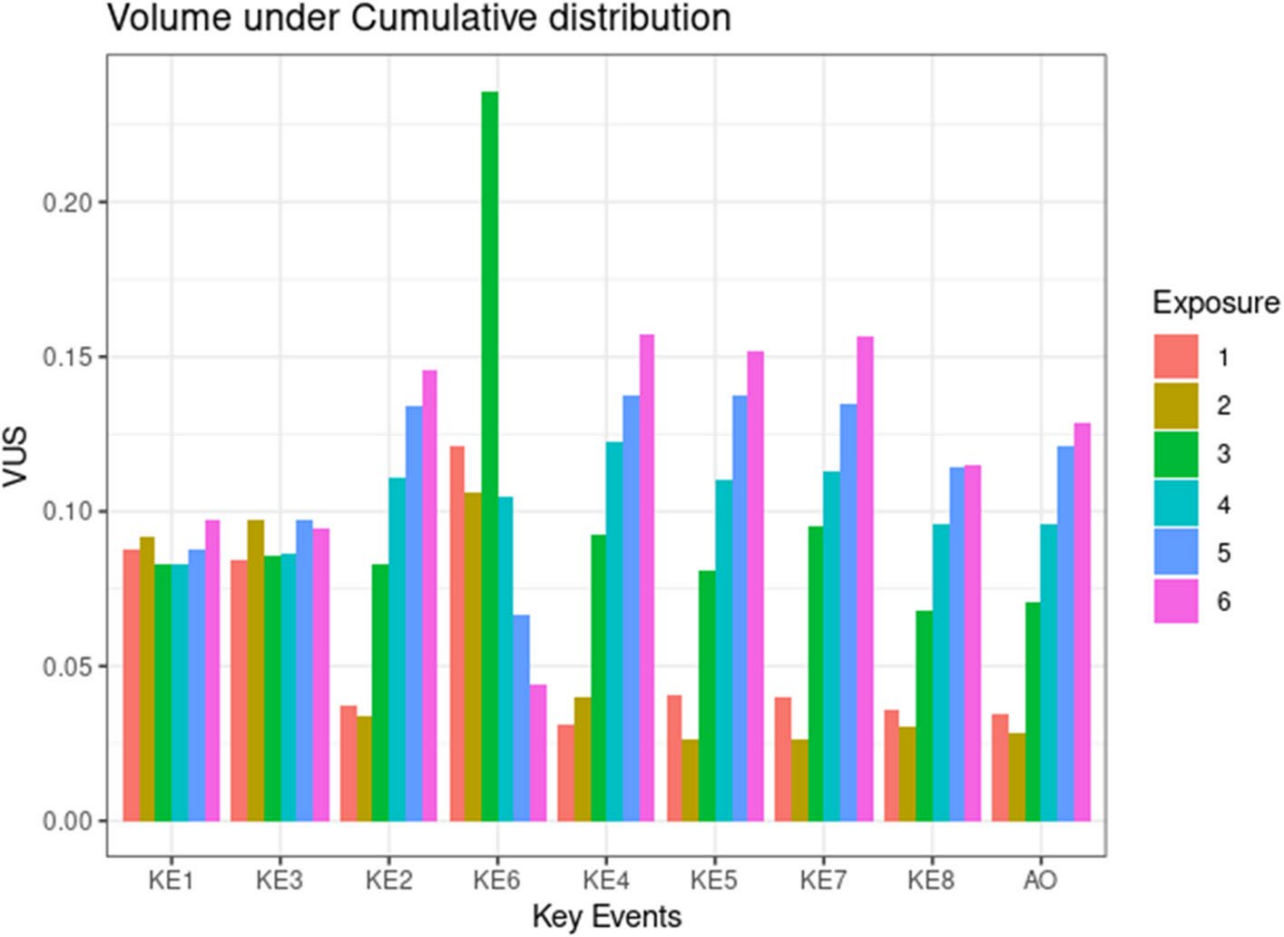
Volume under surfaces of KEs at each exposure repetitions. Probabilities surfaces were calculated by varying the activation threshold $\Delta$ and the dose $d$. The volume under the surface (VUS) of those variables was calculated using trapezoid rule. The analysis and visualization were performed using R statistics software version 4.0.4

GBNs were resampled and the probabilities of KEs at a given dose, $P\left(\nu >\Delta |d\in \left[d\pm \varepsilon \right]\right)$, were estimated using logic sampling. Figure 5 shows these probabilities when the activation threshold was set to $\Delta ={log}_{10}2$. We see that $P\left(KE1>\Delta |d\in \left[d\pm \varepsilon \right]\right)$ increased monotonically with $d$ and showed a strong dose dependence for all exposure repetitions. Similarly, $P\left(KE3>\Delta |d\in \left[d\pm \varepsilon \right]\right)$ also increased but the dose dependence was not as strong as KE1. As expected, probabilities $P\left(\nu >\Delta |d\in \left[d\pm \varepsilon \right]\right)$ for the chronic phase nodes on the other hand were practically zero for $e= 1, 2$, demonstrating that chronic-phase nodes were activated only after a certain exposure repetition. From $e=3$ onwards, $P\left(\nu >\Delta |d\in \left[d\pm \varepsilon \right]\right)$ for all chronic phase nodes increased consistently with successive exposures (${P}_{e=5}\left(\nu >\Delta |d\in \left[d\pm \varepsilon \right]\right)>{P}_{e=4}\left(\nu >\Delta |d\in \left[d\pm \varepsilon \right]\right)$, so and so forth), but did not show a strong dose dependence. Additionally, these probabilities are consistently higher than 30% only from *e* = 5 onwards. The choice of $\Delta ={log}_{10}2$ was adhoc and applied uniformly across all KEs. We also varied $\Delta$ and estimated how probabilities $P\left(\nu >\Delta |d\in \left[d\pm \varepsilon \right]\right)$ varied as a function of both the activation threshold and dose. The plots are shown in Supplementary Fig. 2. A more succinct way of visualizing these surfaces was by calculating the volume under the surface (VUS), $\int P\left(\nu >\Delta |d\in \left[d\pm \varepsilon \right]\right)$. VUSs plot (Fig. 6) showed clearly that the VUSs for two acute phase key events, KE1 & KE3, were comparable across exposure repetitions, highlighting that their activations were exposure repetition independent, but VUSs for chronic phase KEs progressively grew with exposure repetitions post $e=3$, showing once again that chronic phase responses required repeated exposures for their activation (Fig. 6).

While this work was done using virtual data, it showed the kind of information researchers can extract from this type of modeling once in vitro data become available. This work also demonstrates the qAOP formalism is highly flexible and can be used as a tool for prospective study.

### DBN modeling for AO probability calculation

#### General trend pooled dose

**Figure 7:**
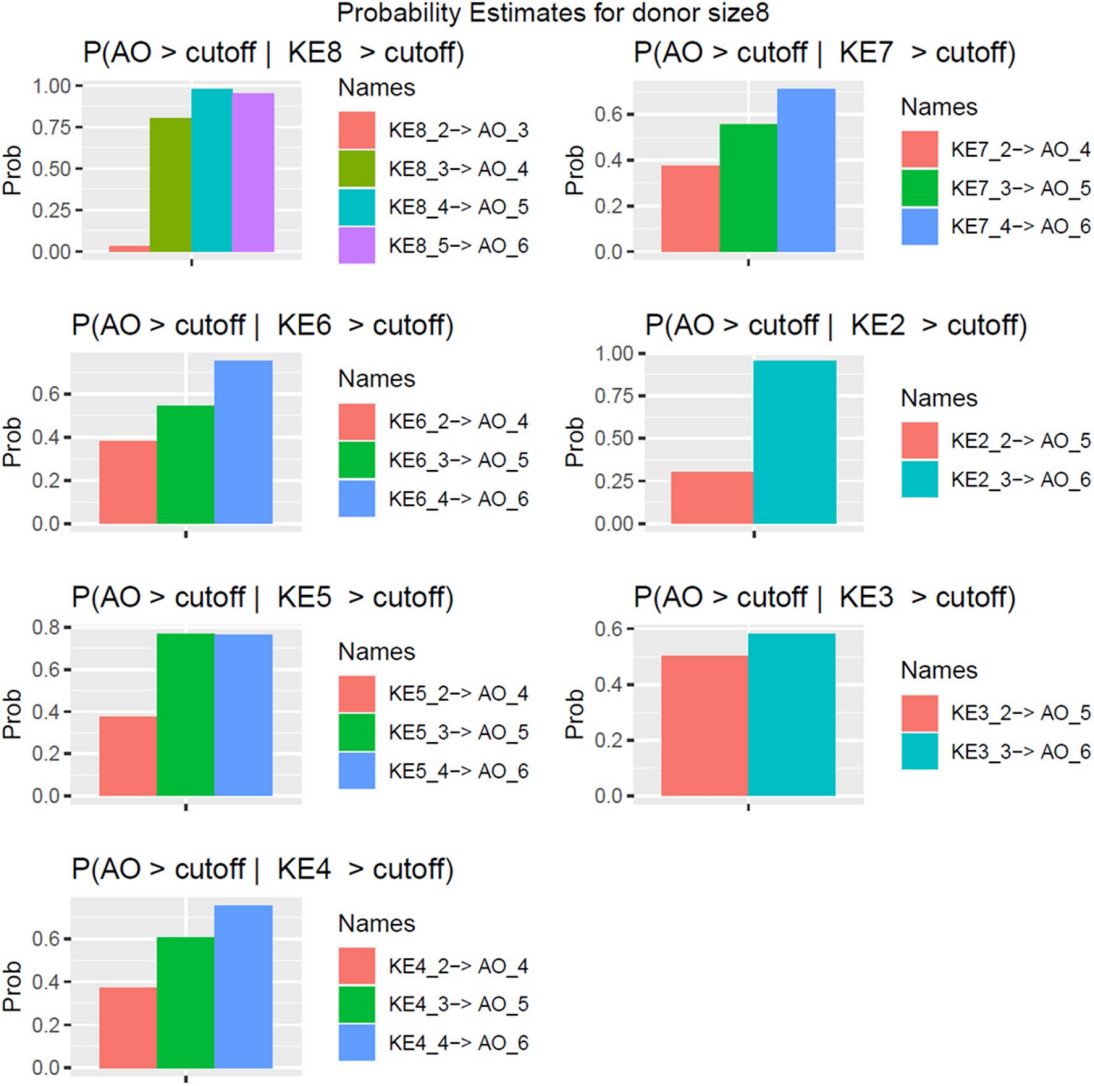
Transition probabilities with all dose groups combined. The transition probabilities were calculated using likelihood weighing after the dynamic Bayesian Network (DBN) was trained with data from successive exposures. The notation $KE\nu \_i \to AO\_j$ was used to denoted $P({AO}^{e=j}>\Delta |{KE\nu }^{e-\tau =i}>\Delta )$**.** The probabilities were calculated when all dose groups were combined. The analysis and visualization were performed using R statistics software version 4.0.4.

Here we explored how an earlier activation of an upstream KE influenced AO subsequently. Formally, we estimated transition probability $P\left({AO}^{\left(e\right)}>\Delta | {KE}^{\left(e-\tau \right)}> \Delta \right)$ by first fitting the DBN (Eq. (8), and Fig. 2) to the data, resampling from the fitted DBNs, and finally estimating the probability using likelihood weighing. For the first part of this work, we pooled all doses together. As shown in the Fig. 2, the arrows causally connected nodes between two successive exposure repetitions. It is worth repeating that unlike time-invariant time-series analysis, the strengths of interactions (the thickness of arrows) between two nodes were not constants, but they changed dynamically with $e$. For example, the strength of interaction $KE8^{{\left( {e - 1} \right)}} \to AO^{\left( e \right)}$ was not the same for $e=3$ and $e=4$: interaction strength was weak for $e=3$ , but strong for $e=4$ and onwards (Fig. 7). We observed that $P\left({AO}^{\left(e\right)}>\Delta | {KE8}^{\left(e-\tau \right)}> \Delta \right)$ (denoted as $KE8^{{\left( {e - \tau } \right)}} \to AO^{\left( e \right)}$ in Fig. 7), increased monotonically as $e \to E = 6$ for all $\tau =1, 2, \& 3$. In addition, activation of KE8 at or after *e* = 4 was highly predictive of adverse outcome (~ 0.96). For KEs that were separated by $\tau =2$, namely KE4, KE5, KE6, & KE7, we found a similar increasing trend: the probability of AO reached values close to 0.8 for $e-\tau =$ 4. For $e-\tau =$ 3, the probability of AO was roughly 0.6. KE3 is an acute-phase response in our model and though it regulated KE7, they were poorly correlated (Fig. 4). Consequently, we did not expect the probabilities $P\left({AO}^{\left(e\right)}>\Delta | {KE3}^{\left(e-3\right)} > \Delta \right), e=\mathrm{5,6}$ to depend on the specific exposure repetition $e$. Our analysis confirmed that indeed $P\left({AO}^{\left(e=5\right)}>\Delta | {KE3}^{\left(e-3=2\right)} > \Delta \right)\cong \left({AO}^{\left(e=6\right)}>\Delta | {KE3}^{\left(e-3=3\right)} > \Delta \right)$ (Fig. 7).

$P\left({AO}^{\left(E\right)}>\Delta | {KE2}^{\left(e=3\right)} > \Delta \right)$, oddly, was very close to 1. The reason remains unclear, but we think that it can be because the outgoing degree (number of outgoing edges) for KE2 is highest among all the chronic KEs.

#### Dose specific trends

**Figure 8:**
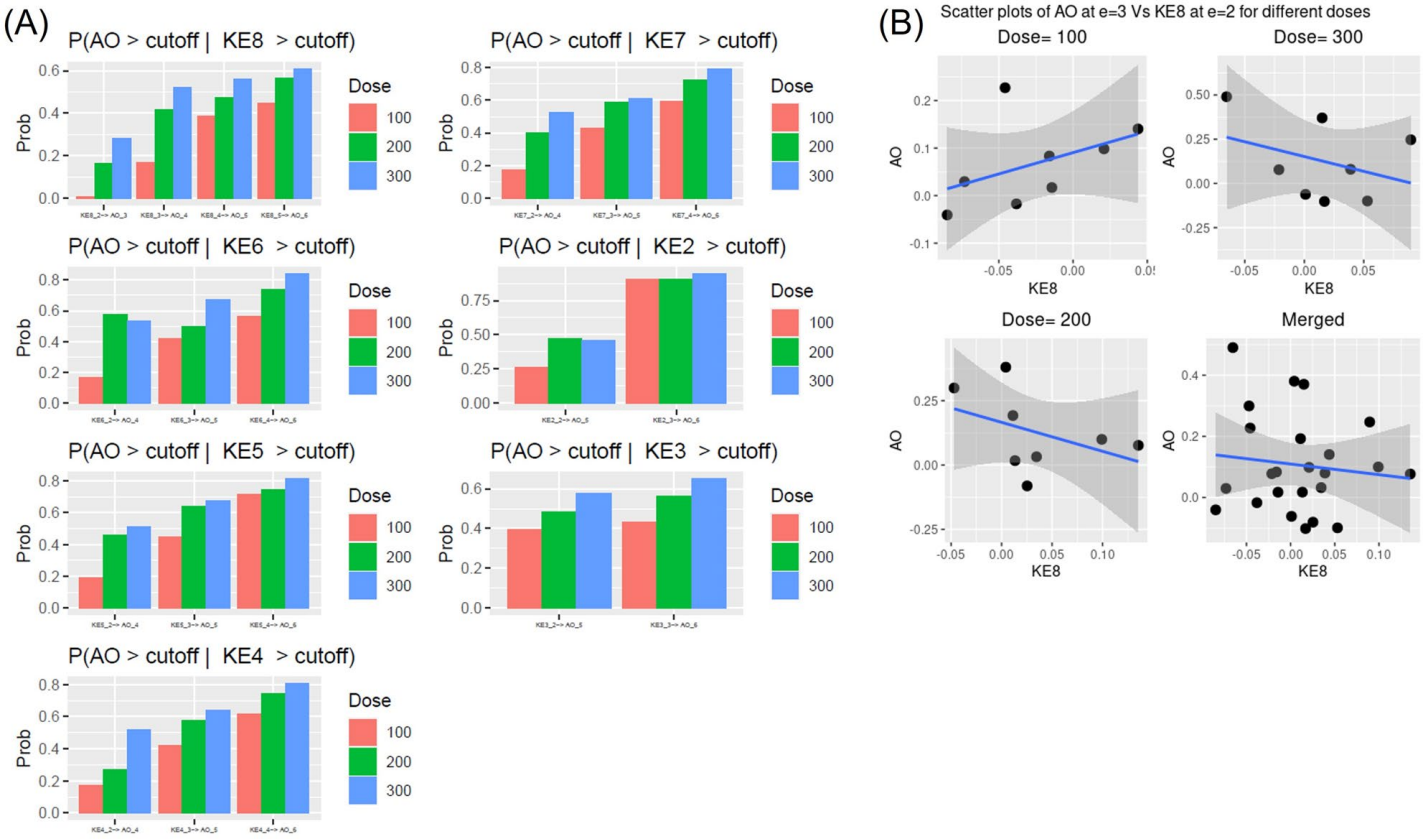
Transition probabilities from each dose separately. (**A**) The transition probabilities were calculated for each dose separately. The dose groups are color coded and like before, $KE\nu \_i \to AO\_j$ stood for $P({AO}^{e=j,d}>\Delta |{KE\nu }^{e-\tau =i,d}>\Delta )$ (**B**) Scatter plots of AO at e = 3 and KE8 at e = 2 at each separate dose and pooled dose. These analyses and visualizations were performed using R statistics software version 4.0.4.

The general trends for dose specific transition probabilities, $P\left({AO}^{\left(e,d\right)}>\Delta | {KE}^{\left(e-\tau ,d\right)}> \Delta \right)$, by-n-large met our expectations: probabilities increased as $e \to E = 6$ for all $\tau =1, 2, 3$, and as the dose $d\uparrow$. Notable exceptions were $P\left({AO}^{\left(e=4,d=200\right)}>\Delta | {KE6}^{\left(e-2=2,d=200\right)}> \Delta \right)$, & $P\left({AO}^{\left(e=5,d=200\right)}>\Delta | {KE2}^{\left(e-3=2,d=200\right)}> \Delta \right)$ (Fig. 8A). One plausible explanation is that the DBNs were fitted with only eight donor level data points for each dose, rendering it error prone. We took a deep dive and visualized log fold changes of ${AO}^{\left(e=3,d\right)}$ vs ${KE8}^{\left(e-1=2,d\right)}$ for all three doses (Fig. 8B). For such an early exposure ($e=3, e-1=2$), chronic responses were not elicited and no correlation between two chronic events was expected. Yet, we found positive correlation for low dose but negative at mid and high dose between AO and KE8. We considered these inconsistent correlations as purely coincidental and artifacts of small sample size.

### Data-driven AOP restructuring

We also developed a data-driven AOP restructuring method based on lasso regression: an interaction was considered important provided pruning that interaction resulted in significant decline in the quality of the fit. In order to accomplish the task of pruning, the GBNs were parameterized using Lasso regression (methods section "Dose–response and response-response analysis"). For nodes with only one parent regulator, we preserved the interaction provided the p-value of the slope parameter was < 0.05.

**Figure 9:**
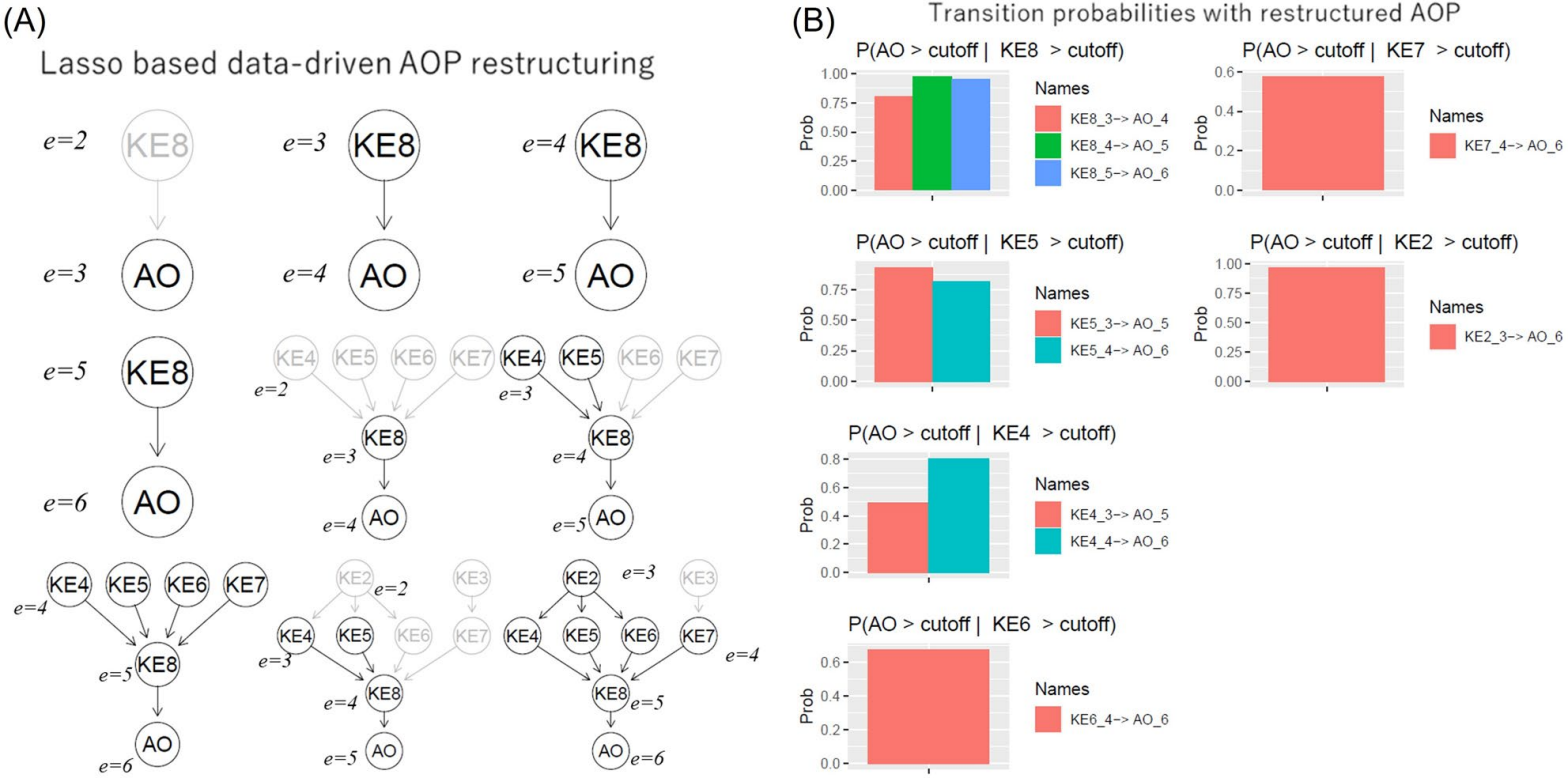
Data-driven AOP restructuring and recalculation of transition probabilities. (**A**) The DBN was trained using lasso regression. The causal links deemed insignificant were shown in grey. (**B**) Probabilities were calculated using likelihood weighing, and only those transition probabilities where $P\left({AO}^{\left(e\right)}>\Delta |{KE}^{\left(e-\tau \right)}>\Delta \right)\ne P\left({AO}^{\left(e=3\right)}>\Delta \right)$, were shown. Like before, $KE\nu \_i \to AO\_j$ was used to denoted $P({AO}^{e=j}>\Delta |{KE\nu }^{e-\tau =i}>\Delta )$**.** These analyses and visualizations were performed using R statistics software version 4.0.4.

In the repeated dosing study, it could be expected that the strength of causal links varied based on exposure repetition. In addition, changes in the causal relationship could result in some of the predictors to be uninformative. The data-driven pruning of DBNs are shown in Fig. 9A. The uninformative predictors and their links are shown in grey. We found that KE8 at *e* = 2 did not exert any influence on AO at *e* = 3: the probability of AO at *e* = 3 was conditionally independent of KE8, i.e. $P\left({AO}^{\left(e=3\right)}>\Delta |{KE8}^{\left(e=2\right)}>\Delta \right)=P\left({AO}^{\left(e=3\right)}>\Delta \right)$. For all subsequent exposure slices, KE8 exerted influence on AO outcomes. Similarly, we found KE4, KE5, KE6, and KE7 at *e* = 2 had no bearing on AO at *e* = 4; KE4 and KE5 at *e* = 3 conditionally influenced AO at *e* = 5. All these KEs were conditional determinants of AO only for *e* = 6. KE3 was never a conditional determinant, and KE2 at *e* = 3 influenced AO at *e* = 6 but not for any earlier time slices. This data-driven remodeling enabled us to reevaluate the transition probabilities and extract only the relevant conditional probabilities ($P\left({AO}^{\left(e\right)}>\Delta |{KE}^{\left(e-\tau \right)}>\Delta \right)\ne P\left({AO}^{\left(e=3\right)}>\Delta \right)$), as shown in Fig. 9B. We found that while the numerical values of transition probabilities were a little different from the DBN with whole AOP structure, their qualitative characteristics were still the same: $P\left({AO}^{\left(e\right)}>\Delta |{KE8}^{\left(e-1\right)}>\Delta \right)$ was very high for $e\ge 4$; $P\left({AO}^{\left(e\right)}>\Delta |{KE5}^{\left(e-2\right)}>\Delta \right)$ was $\succsim$ 0.8 for $e=5$ but went down to ~ 0.7 for $e=6$; $P\left({AO}^{\left(e\right)}>\Delta |{KE4}^{\left(e-2\right)}>\Delta \right)$ increased from 0.5 for $e=5$ to ~ 0.8 for $e=6$; $P\left({AO}^{\left(e\right)}>\Delta |{KE6}^{\left(e-2\right)}>\Delta \right)$ was ~ 0.8 for $e=6$ and it was the only instance when AO is conditionally dependent of KE6; $P\left({AO}^{\left(e\right)}>\Delta |{KE7}^{\left(e-2\right)}>\Delta \right)$ was ~ 0.6 for $e=6$; $P\left({AO}^{\left(e\right)}>\Delta |{KE2}^{\left(e-3\right)}>\Delta \right)$ ~ 1 for $e=6$. A plausible reason for this high value was discussed in the previous section.

## Discussion

The goal of the AOP framework is to provide a principled approach to better understand the pathway to adverse outcome by synergistically integrating information from different layers of biological organization and advance a more informed testing strategy^3^. Owing to its flexibility and its ability to ingest information, not just from in vivo animal models but also from in vitro new approach methodologies, the AOP framework has emerged as a powerful tool for chemical testing and risk assessment. Cost effective in vitro methods enable toxicologists to collect information from different layers of biological organizations that can then be integrated into a cohesive AOP framework and used as a roadmap for an adverse outcome. Over the years, the number of expert derived AOP submissions have grown (https://aopwiki.org/). There are several AOPs that deal in chronic toxicity as the AO, however, AOPs that deal with repeated exposure and donor dependent onset of chronic events are still sparse. One reason for this humble showing can be difficulty in AOP to represent dynamism of biological pathway perturbation during repeated exposure to stimuli. Regardless, dearth of repeated exposure related AOPs that deal with acute phase and chronic phase KEs differently, account for donor dependent onset of chronic events, needs to be addressed.

Also, generation of the data alone is not sufficient. Data arising from molecular to phenotypic level need to be integrated and interpreted unambiguously to really render an AOP useful. Moreover, the associations between different layers of biological organization need to be quantified and made manifest. How these associations evolve over different exposure repetitions need to be quantified. Only a principled quantitative approach can achieve that.

To demonstrate the utility of a chronic qAOP framework by taking repeated exposure into consideration, we used a hypothetical AOP with acute phase responses (KEs and BMs), chronic phase KEs, and AO. As we mentioned, BMs are involved in this hypothetical AOP, because some secondary measures outside AOP could be also obtained in the real experimental situations, and they could be informative of AOP perturbation. Separation of acute phase KEs and chronic phase KEs/AO enabled us to consider the dynamism of biological perturbation. We generated a virtual dataset to quantify this AOP. In generating this dataset, we assumed that the chronic KEs were initiated only after repeated insults whereas the acute KEs were readily activated. In this study, we assumed a six-time repeated exposure study as a proof of concept, although user-defined study design is acceptable for our model. The acute KEs showed strong dose–response, the chronic KEs showed dose–response and exposure repetition-response only after they were activated. We further assumed that the onset of chronic KEs varied from donor to donor, as in vitro models with which the researcher can perform repeated exposure study is often composed of primary cells. Primary cells generally possess their original characteristics, could thus show donor-dependent variation in response against the same stimuli^27–29^.

Using a GBN, we showed that several pertinent questions regarding probability of an adverse event can be answered quantitatively and qAOP model can be used prospectively in risk assessment. Specifically, using GBN model we calculated probabilities of KEs and AO to be activated above a certain threshold $\Delta$ given a dose $d$ and an exposure repetition $e$. We varied $\Delta$ and $d$ and investigated how the probability of KEs change. While what constitutes a threshold is debatable and subjective, on quick way to estimate $\Delta$ can be from the dose–response curve. The point here is, even when $\Delta$ is unknown, the BN framework gives us the flexibility to prospectively study the effect of varying $\Delta$ on probabilities of KEs and AOs. Therefore, we implemented the function to depict the changes in the probability as three-dimensional plot. The volume under the cumulative distribution (i.e., volume under the probability surface, VUS) can be then calculated as an index of the AOP perturbation with varied $\Delta$(Fig. 6). The result of VUS calculation demonstrates that the acute KEs and chronic KEs behave very differently: VUS of acute KEs do not change significantly with exposure repetitions, whereas VUS for chronic KEs show strong exposure repetition dependence. The point to note is that this proof-of-concept study utilizes virtually generated data; real biological samples may not be as conclusive. Most important point here is that our approach enables to formulate the different types of analysis, as the model demonstrates what was intuitively clear: probability of chronic phase outcomes increase with repeated exposure.

We also used the DBN and the repeated exposure data to estimate probability of AO to be activated when a certain KE was activation earlier (transition probability). We showed that activation of chronic KEs earlier can be used as a reliable proxy for AO activation later. Not surprisingly, the closer the proximity of the KE to the AO node, the better predictor it was of AO activation. We also showcased what can happen when acute phase events are poorly correlated with chronic events. Such information bottleneck, when present, can seriously limit the ability to infer likelihood of AO based on early evidence of acute phase response. As clear from Figs. 7 and 8A (P(AO > cut off | KE2 > cut off), inference of AO can be compromised when based solely on upstream events that are information bottlenecks. We further demonstrated that these transition probabilities also showed a clear dose dependence. Furthermore, we proposed data-driven restructure of AOP where uninformative KEs could be eliminated from the probability calculations. This approach would help in reducing calculation cost and complexity of AOP on repeated-exposure-oriented chronic toxicity. Using lasso-based pruning, we demonstrate that the wiring in an AOP is itself dynamic, changing over time (Fig. 9). Such flexibility is well known in biology and must be taken into account when drawing inference on AO. Here we showed clearly that the DBN method can be used in detection of early events that are strong proxies for AO. DBN formalism is extremely flexible and very pertinent for biology in general because it can model feedback loops that are ubiquitous in any biological processes. While we fully acknowledge that we did not incorporate all the potential confounders of a real dataset, we believe that our in silico approach can serve as a proof-of-concept and bring home the fact that several specific questions regarding an adverse event, including dose and early response dependent probability of adverse outcome can be answered quantitatively.

## Conclusion

Although several reports attempted to model chronic toxicity^13,30^, they mostly stand upon a simple sequence from MIE through AO, in which cumulative effects are not modeled. We here demonstrated BN and DBN formalisms could enable us to incorporate cumulative effect as well as repeated perturbation of biological pathway. This means that the AOP structure itself could become dynamic as KEs can be annotated as acute or chronic phase. Although it should be acknowledged that our model was developed with a virtual setup (i.e., a hypothetical AOP with a virtual dataset), we believe our proof-of-concept study was able to explore the possibility that AOP framework could be expanded for more flexible toxicity consideration. In addition, considering a part of overlap between chronic toxicity and diseases, our concept for AOP framework could be the future application for the risk assessment of chronic diseases if relevant in vitro NAMs and AOPs are available.

The current study utilizes a hypothetical AOP with two MIEs. Usually, AOPs do not have multiple MIEs. The reason for us to have two MIEs are really two folds. First, multiple assays can be used to measure an MIE. While it is entirely feasible to subsume all these endpoints, nothing in principle prevents them from being treated separately for modeling. Secondly, having multiple MIEs in our framework enables this method to be seamlessly translated to an AOP-network scenario, where multiple MIEs can influence a downstream KE^31^.

In addition, using high throughput screening assays and high content imaging, molecular and phenotypic endpoints can be captured more intensively than ever. AI and machine learning (ML) approaches with these data stream could be highly compatible with AOP framework. So far, inference of outcomes by stimulation is largely dependent on molecular level information thus limited to the early and apical outcomes. However, AOPs provide a natural framework for integration of molecular, phenotypic and organ level information. qAOPs—quantified using HTS, HC imaging, and other NAM data—can be used for estimation of AO conditioned on early evidence. Here we showed two such scenarios in Fig. 5 and Sup Fig. S2. In principle, evidence of arbitrary complexity can be modeled. For prospective study, qAOP can be used to simulate perturbation, and test alternate hypothesis. qAOP wirings can be altered in silico, and various scenarios that can potentially decrease likelihood of AO can be explored. Furthermore, and most importantly, qAOPs can be used for data poor chemical as a read-across by considering qAOPs of structural analogs.

For the practical implementation of NAMs, including qAOP models, within the regulatory framework, several key points need to be addressed. It is crucial to promote the evolution of science through knowledge sharing by reusing both input and output data across different studies. Therefore, the use of a common reporting template is essential^32^. Additionally, the modeling approach should always be evaluated in terms of its domains of applicability, potential bias, and level of certainty or uncertainty^33^.

When focusing on qAOP modeling, transparency in the modeling approach, dataset, and their quality and reliability are of utmost importance for decision-making^34^. To this end, guidelines and workflows have already been proposed, and researchers are encouraged to follow them when using the qAOP models including the models developed in this study. By adhering to these practices, we can ensure greater consistency and reliability in the application of qAOP models in regulatory decision-making processes.

### Supplementary Information

Supplementary Information 1.

Supplementary Information 2.

Supplementary Table 3.

Supplementary Figure 4.

Supplementary Figure 5.

## Data Availability

The data that support the findings of this study are available from the corresponding author, upon reasonable request.

## Abbreviations

AOP: Adverse outcome pathway
NAMs: New approach methodologies
BN: Bayesian network
DBN: Dynamic Bayesian network
GBN: Gaussian Bayesian network
DAG: Directed acyclic graph
MIE: Molecular initiating event
KE: Key event
AO: Adverse outcome
BM: Biomarker
LOOCV: Leave-one-out cross validation
VUS: Volume under surface
