# Supplementary material for: Proof of concept for quantitative adverse outcome pathway modeling of chronic toxicity in repeated exposure
Source: Sci Rep. 2024 Feb 27;14:4741. doi: 10.1038/s41598-024-55220-4 (PMC10899215; doi:10.1038/s41598-024-55220-4)
Supplement: Supplementary file 1 — Supplementary Information 1. [file 41598_2024_55220_MOESM1_ESM.docx]

**Title**

Proof of Concept for Quantitative Adverse Outcome Pathway Modeling of Chronic Toxicity in Repeated Exposure.

**Authors**

Shigeaki Ito^1*^, Sayak Mukherjee^2^, Kazuo Erami^1^, Shugo Muratani^1^, Akina Mori^1^, Sakuya Ichikawa^1^, William White^2^, Kei Yoshino^1^, Dawn Fallacara^2^

**Author affiliations**

1. Scientific Product Assessment Center, Japan Tobacco Inc., 6-2, Umegaoka, Aoba-ku, Yokohama, Kanagawa, 227-8512, Japan.

2. Battelle, 505 King Ave., Columbus, OH 43201

* To whom correspondence should be addressed at Scientific Product Assessment Center, Japan Tobacco Inc., 6-2, Umegaoka, Aoba-ku, Yokohama, Kanagawa, 227-8512, Japan. Phone: 81-70-1069-7124; E-mail: [shigeaki.ito@jt.com](mailto:shigeaki.ito@jt.com)

**Supplementary Material 1 Algorithm of each calculation and R packages.**

We used freely downloadable software package called [bnlearn](https://www.bnlearn.com/) to perform all our analysis. We used it mostly for parameter learning, and re-sampling using logic sampling algorithms. Bnlearn allows user to also specify pre-defined parameter values using a function called custom.fit. We used that function when needed. Below, we share the pseudo-codes used for our work. All the codes were written in R version 4.04. We refer readers to section 2.1 of the main text for notation convention

**Algorithm BN update: Train GBN using posterior from previous exposure and re-sample**

$$\mathcal{D ⟵\emptyset}$$

$$for n=1,..,.,N$$

$$for d \in D$$

$$for e=1,.,.,E$$

Load $\boldsymbol{\mu}^{\left( n,e,d \right)},\mathbb{O}_{\left| V \right|\times\left| V \right|}^{\left( n,e,d \right)}$ from the saved library

Draw $\kappa$ samples ${\mathbb{X}_{\kappa\times\left| V \right|}}^{(n,e,d)}\mathcal{\sim MN}\left( \boldsymbol{\mu}^{\left( n,e,d \right)},\mathbb{O}_{\left| V \right|\times\left| V \right|}^{\left( n,e,d \right)} \right)$, and mean center the data

$for v \in V_{\neq rn}\subset V$ (where $V_{\neq rn}$ denotes set of all nodes except the root nodes)

Calculate ${\hat{\boldsymbol{\beta}}}_{\boldsymbol{OLS}}$ & $\hat{\sigma}^{2}$ (see section 2.6 main text)

If $e=1$

$\begin{matrix} {\boldsymbol{\beta}^{\boldsymbol{(}e\boldsymbol{)}}}_{pos}={\hat{\boldsymbol{\beta}}}_{\boldsymbol{OLS}} \\ {\mathbb{V}^{(e)}}_{pos}=\left( {\mathbb{X}_{{\kappa\times\Pi}_{v}}}^{T}\mathbb{X}_{{\kappa\times\Pi}_{v}} \right)^{\boldsymbol{-1}}\hat{\sigma}^{2} \end{matrix}$

else

${\boldsymbol{\beta}^{\boldsymbol{(}e\boldsymbol{)}}}_{pr}\boldsymbol{,}{\mathbb{V}^{(e)}}_{pr}={{\hat{\boldsymbol{\beta}}}^{(e-1)}}_{pos}\boldsymbol{,}{\mathbb{V}^{(e-1)}}_{pos}$ (see section 2.6 main text)

Store posteriors in a list

$$for n=1,.,.,N$$

$$for d \in D$$

$$for e=1,.,.,E$$

Topologically order node based on **Fig. 1 main text**

Draw $\mathcal{R}$ replicates for the root nodes $\in V_{rn} \subset V$; ${\mathbb{X}_{\mathcal{R\times}\left| V_{rn} \right|}}^{(n,e,d)}\mathcal{\sim MN}\left( {\boldsymbol{\mu}_{\left| \boldsymbol{V}_{\boldsymbol{rn}} \right|}}^{\left( n,e,d \right)},\mathbb{O}_{\left| \boldsymbol{V}_{\boldsymbol{rn}} \right|\times\left| \boldsymbol{V}_{\boldsymbol{rn}} \right|}^{\left( n,e,d \right)} \right)$ and mean center it.

Using GBN shown in **Fig. 1 main text** and topological ordering, predict ${\boldsymbol{x}^{\boldsymbol{(}\nu\boldsymbol{)}}}_{pred}\sim P\left( {\boldsymbol{x}^{\boldsymbol{(}\nu\boldsymbol{)}}}_{pred}|\mathbb{X}_{\mathcal{R\times}\left| \Pi_{v} \right|}^{(n,e,d)},\sigma^{2}\mathbb{I} \right)$ for all downstream nodes (see section 2.6)

$\mathcal{D ⟵}$append ($\mathcal{D,}{\boldsymbol{x}^{\boldsymbol{(}\nu\boldsymbol{)}}}_{pred}+\mu^{\left( n,e,d,v \right)}\mathbb{1}$)

Output $\mathcal{D}$ in desired format $\mathbb{X}_{out}$

**Algorithm: Bayesian Network analysis**

$$for e=1, 2, ., E$$

${GBN}^{e}=bn.fit\left( Fig. 1,\mathbb{X}^{\left( e \right)} \right)$ (uses linear regression to learn parameters)

$$for \nu in KEs \subset V$$

Cutoff_arr = $seq\left( from=mean\left( \boldsymbol{x}^{\left( e,v \right)} \right),to=max\left( \boldsymbol{x}^{\left( e,v \right)} \right),by=bw \right)$

$$for d \in D$$

Calculate $\xi_{d}^{\left( \nu,e \right)}=P\left( \nu>\log_{10} 2 | d\in\left[ d\pm\varepsilon\right] \right),$using logic sampling ($\varepsilon$ is the tolerance)

$for c in$Cutoff_arr

Calculate ${Surf}_{c,d}^{\left( \nu,e \right)}=P\left( \nu>c | d\in\left[ d\pm\varepsilon\right] \right)$ using logic sampling.

Calculate ${VUS}^{\left( \nu,e \right)}=\iint{Surf}_{c,d}^{\left( \nu\right)}$

Return ${VUS}^{\left( \nu,e \right)}$, $\boldsymbol{\xi}^{\left( \nu,e \right)}$

**Algorithm: Dynamic Bayesian Model for probability of AO based on activation of KEs upstream KEs**

Inputs:

- Inference Node=AO
- Start point =$e_{s}$
- Number of time slices $\tau$

If $e_{s}$ < 1 or $e_{s} + \tau> E$ print(“Mistake”) & quit

$Exp.seq⟵$ $seq\left( from=e_{s}+\tau, to=e_{s}+1,by=-1 \right)$

$C.nd$ $⟵$ AO

$r.nd$ $⟵$ root.nodes (DAG in Fig. 1 main text)

$r.nd.visit.time\leftarrow\emptyset$ (a list or dictionary with nodes as keys)

$$for e in Exp.seq$$

$$tmp ⟵ \emptyset$$

$if\left( C.nd\cap r.nd\neq\emptyset\right)$

$$for \nu in C.nd\cap r.nd$$

$$r.nd.visit.time\left[ \nu\right]\leftarrow append\left( r.nd.visit.time\left[ \nu\right],e \right)$$

$$for \nu in \left( C.nd-C.nd\cap r.nd \right)$$

tmp $⟵$append(tmp, $\Pi_{\nu}$)

If $\left| \Pi_{\nu} \right| > 1$

fit ridge model $cv.glmnet\left( y=\boldsymbol{x}^{\left( e,\nu\right)},x=\mathbb{X}^{\left( e-1,\Pi_{\nu} \right)}, \alpha=0,n.flds=LOOCV \right)$

else

fit linear regression $\boldsymbol{x}^{\left( e,\nu\right)}=\beta_{0}\mathbb{1}+\beta_{1}\boldsymbol{x}^{\left( e-1,\nu\right)}+\boldsymbol{\varepsilon}\sim N\left( 0,\sigma^{2}\mathbb{I} \right)$ with LOOCV using caret package

$C.nd$ $⟵$ unique(tmp)

If ($C.nd = r.nd$) break

$$e^{*}=e-1$$

$$for \nu in C.nd$$

$\boldsymbol{x}^{\left( e^{*},\nu\right)}\sim\beta_{0}+\varepsilon\sim N\left( 0,\sigma^{2} \right)$ , where $\beta_{0}=mean\left( \boldsymbol{x}^{\left( e^{*},\nu\right)} \right)$ & $\sigma=sd\left( \boldsymbol{x}^{\left( e^{*},\nu\right)} \right)$

If ($r.nd.visit.time\neq\emptyset$)

$$for \nu in r.nd.visit.time$$

$$fore^{*} in r.nd.visit.time\left[ \nu\right]$$

$\boldsymbol{x}^{\left( e^{*},\nu\right)}\sim\beta_{0}+\varepsilon\sim N\left( 0,\sigma^{2} \right)$ , where $\beta_{0}=mean\left( \boldsymbol{x}^{\left( e^{*},\nu\right)} \right)$ & $\sigma=sd\left( \boldsymbol{x}^{\left( e^{*},\nu\right)} \right)$

Customize BN using the fitted parameters and custom.fit function in bnlearn

Return customized BN

**Algorithm: Pruning based on WoE of DBNs (changes from the previous algorithm are highlighted)**

Inputs:

- Inference Node=AO
- Start point =$e_{s}$
- Number of time slices $\tau$

If $e_{s}$ < 1 or $e_{s} + \tau> E$ print(“Mistake”) & quit

$Exp.seq⟵$ $seq\left( from=e_{s}+\tau, to=e_{s}+1,by=-1 \right)$

$C.nd$ $⟵$ inference.node

$r.nd$ $⟵$ root.nodes (DAG in Fig. 1 main text)

$$r.nd.visit.time\leftarrow\emptyset$$

$$for e in Exp.seq$$

$$tmp ⟵ \emptyset$$

$if\left( C.nd\cap r.nd\neq\emptyset\right)$

$$for \nu in C.nd\cap r.nd$$

$$r.nd.visit.time\left[ \nu\right]\leftarrow append\left( r.nd.visit.time\left[ \nu\right],e \right)$$

$$for \nu in \left( C.nd-C.nd\cap r.nd \right)$$

tmp $⟵$append(tmp, $\Pi_{\nu}$)

If $\left| \Pi_{\nu} \right| > 1$

fit lasso model $cv.glmnet\left( y=\boldsymbol{x}^{\left( e,\nu\right)},x=\mathbb{X}^{\left( e-1,\Pi_{\nu} \right)}, \alpha=1,n.flds=LOOCV \right)$

only include predictors $\Pi_{\nu}^{'}\subset\Pi_{\nu}$ whose lasso coefficients are non-zero

else

fit linear regression $\boldsymbol{x}^{\left( e,\nu\right)}=\beta_{0}\mathbb{1}+\beta_{1}\boldsymbol{x}^{\left( e-1,\nu\right)}+\boldsymbol{\varepsilon}\sim N\left( 0,\sigma^{2}\mathbb{I} \right)$ with LOOCV using caret package

if p.value($\beta_{1}$) > 0.05

set $\beta_{1}=0$

$C.nd$ $⟵$ unique(tmp)

If ($C.nd = r.nd$) break

$$e^{*}=e-1$$

$$for \nu in C.nd$$

$\boldsymbol{x}^{\left( e^{*},\nu\right)}\sim\beta_{0}+\varepsilon\sim N\left( 0,\sigma^{2} \right)$ , where $\beta_{0}=mean\left( \boldsymbol{x}^{\left( e^{*},\nu\right)} \right)$ & $\sigma=sd\left( \boldsymbol{x}^{\left( e^{*},\nu\right)} \right)$

If ($r.nd.visit.time\neq\emptyset$)

$$for \nu in r.nd.visit.time$$

$$fore^{*} in r.nd.visit.time\left[ \nu\right]$$

$\boldsymbol{x}^{\left( e^{*},\nu\right)}\sim\beta_{0}+\varepsilon\sim N\left( 0,\sigma^{2} \right)$ , where $\beta_{0}=mean\left( \boldsymbol{x}^{\left( e^{*},\nu\right)} \right)$ & $\sigma=sd\left( \boldsymbol{x}^{\left( e^{*},\nu\right)} \right)$

Customize BN using the fitted parameters and custom.fit function in bnlearn

Return customized BN
