# Supplementary material for: Proof of concept for quantitative adverse outcome pathway modeling of chronic toxicity in repeated exposure
Source: Sci Rep. 2024 Feb 27;14:4741. doi: 10.1038/s41598-024-55220-4 (PMC10899215; doi:10.1038/s41598-024-55220-4)
Supplement: Supplementary file 5 — Supplementary Figure 5. [file 41598_2024_55220_MOESM5_ESM.docx]

**Title**

Proof of Concept for Quantitative Adverse Outcome Pathway Modeling of Chronic Toxicity in Repeated Exposure.

**Authors**

Shigeaki Ito^1*^, Sayak Mukherjee^2^, Kazuo Erami^1^, Shugo Muratani^1^, Akina Mori^1^, Sakuya Ichikawa^1^, William White^2^, Kei Yoshino^1^, Dawn Fallacara^2^

**Author affiliations**

1. Scientific Product Assessment Center, Japan Tobacco Inc., 6-2, Umegaoka, Aoba-ku, Yokohama, Kanagawa, 227-8512, Japan.

2. Battelle, 505 King Ave., Columbus, OH 43201

* To whom correspondence should be addressed at Scientific Product Assessment Center, Japan Tobacco Inc., 6-2, Umegaoka, Aoba-ku, Yokohama, Kanagawa, 227-8512, Japan. Phone: 81-70-1069-7124; E-mail: [shigeaki.ito@jt.com](mailto:shigeaki.ito@jt.com)

**Supplementary figure 2: Cumulative distribution plot at each exposure repetition.**

The probabilities were calculated by fitting the GBN to the virtual data by varying activation threshold Δ and the dose d. The data are represented as cumulative distribution plot.
